# Transjugular liver biopsy: What to do and what not to do

**DOI:** 10.4103/0971-3026.41839

**Published:** 2008-08

**Authors:** Shyamkumar N Keshava, Thomas Mammen, NRS Surendrababu, Vinu Moses

**Affiliations:** Department of Radiology, Christian Medical College, Vellore - 632 004, Tamil Nadu, India

**Keywords:** Transjugular liver biopsy

The presence of ascites or deranged bleeding parameters in patients with significant liver parenchymal disease precludes percutaneous liver biopsy (PLB). Transjugular liver biopsy (TJLB) is an important and safer alternative to the traditional method of PLB. This article highlights the technical aspects of the procedure and describes the standard way of performing the procedure, the common problems encountered, and the ways of overcoming them.

A transjugular approach to reach the liver was first described by Weiner *et al.* Transjugular liver biopsy (TJLB)[1,2] is a technique for obtaining liver biopsy without causing a transcapsular injury. Using a transvenous approach, the biopsy needle is inserted into the liver via the hepatic vein, avoiding the peritoneum and the liver capsule. Thus, if there is any bleeding related to the procedure, it will be back into the venous system. Earlier, when aspiration needles[3] were used for biopsy, the quality of the specimen was considered to be inferior to that obtained by percutaneous liver biopsy (PLB). With the advent of the Tru-Cut (Quick-Core biopsy needle, Cook, Bloomington), TJLB specimens have been found to be of the same quality as the PLB specimens.[4–7]

## Patient selection

TJLB is performed in patients when liver biopsy is essential for the diagnosis and management but percutaneous biopsy is contraindicated due to deranged bleeding parameters or ascites.

Indications:

1. Ascites
2. Coagulopathy (prolonged PT or PTT or a low platelet count) to the extent that PLB is contraindicated. The degree of coagulopathy at which PLB is contraindicated varies with different institutions; a safe upper limit for deranged bleeding parameters has not been described in the literature. In our institution, additional blood products are given if the International Normalized Ratio (INR) is >1.7 times the control and the platelet count is <35,000.
3. Other reasons, e.g., a small shrunken liver, extreme obesity, or peliosis hepatis.

## TJLB procedure

Setup and materials required:

- Angiography suite with ultrasound machine
- TJLB set (LABS-100, Cook), 9F short sheath (11 cm), catheters (multipurpose, cobra, and headhunter), and guide wires (0.035 inch guide wire and stiff glide wire)

### Patient preparation (ward/day care admission)

TJLB is performed as an inpatient procedure. USG of the abdomen is performed prior to the procedure to assess liver size, identify any focal lesion and, if possible, to assess the hepatic veins. Fasting for 4 - 6 h prior to the procedure is advisable to reduce the chances of aspiration. The patient may be sedated before the procedure.

### Getting the patient ready for the procedure in the angiography room

Patients have to be in the supine position and should be relatively comfortable. The operators work from the head end of the patient. USG is used to confirm the patency of the right jugular vein. If real-time USG guidance is not available, then the internal jugular vein (IJV) should be marked on the skin. A point 3 - 5 cm above the clavicle is selected for the puncture. If the site of access is too close to the clavicle, the chances of inadvertent pleural puncture may be higher. If necessary, the patient's head can be turned to the contralateral side. Asking the patient to do a Valsalva maneuver or elevating the foot end of table can help in distending the IJV, if necessary.

Patient monitoring with ECG, BP, and pulse oximeter is important during the procedure.

### Obtaining venous access [Figure 1]

**Figure 1:**
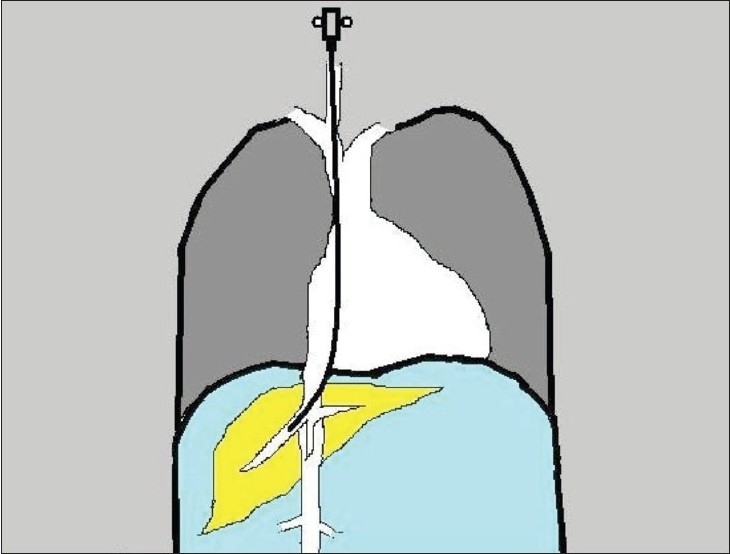
A line diagram showing the orientation of the TJLB needle

The patient's neck is cleaned and draped using sterile precautions. Local anesthetic (2% lignocaine; 2 - 5 ml) is infiltrated intradermally and subcutaneously at the site of puncture. The right IJV (single wall) is then punctured under USG guidance, using an 18G metal needle with a plastic cannula (Insyte, BD) or an 18G metallic needle. A micropuncture (21G) needle is used for children and in patients with grossly deranged bleeding parameters. A syringe must be connected to the needle for aspiration of blood and also to prevent air embolism. Aspirating venous blood will help in confirming the jugular puncture. Caution should be exercised to avoid carotid puncture and pneumothorax. When the right IJV is not accessible, the left IJV[8] or femoral vein[9] can act as alternative venous access sites.

Once the vein is punctured, a suitable guide wire (0.035 inch for 18G) is passed through it, followed by placement of a 9-French sheath.

### Negotiation into the right hepatic vein (RHV)

The wire needs to be negotiated along the SVC-right atrium-IVC and into the RHV. A combination of 5-Fr multipurpose catheter and guide wire (0.035 inch J tip or straight tip) is used to navigate from the SVC through the IVC and into the RHV.

Arrhythmias should be watched for during the transit through the right atrium. Such arrhythmias are usually transient and subside once the catheter/wire reaches the IVC. Care must be taken to ensure that the catheter/wire does not form any loops in the right atrium.

If there is any difficulty in crossing the right atrium, the following can be tried:

- Use of different guide wires, like J tip or straight tip, or a glide wire
- Attempt to pass the wire in different phases of respiration (e.g., deep inspiration)
- Change the angle of the fluoroscopy image intensifier into steep anterior oblique and direct the catheter posterolaterally from the right atrium into the IVC

Negotiating the wire or catheter into the RHV from the IVC can sometimes be challenging. The RHV is usually located just caudal to the inferior cavo-atrial junction, with all three hepatic veins being at the same level. If any difficulty is encountered in accessing the RHV with the multipurpose catheter, the following can be tried:

- Attempt to pass the wire in different phases of respiration (e.g., deep inspiration).
- The RHV may not be in the true coronal plane and hence minimal anterior or posterior angulations can be tried at the expected cranio-caudal level.
- Different catheters like headhunter or cobra catheter and a 0.035 inch glide wire can be used instead of the regular guide wire.
- If the level of the hepatic veins cannot be assessed, a 5F pigtail run is obtained near the cavo-atrial junction. Filling of hepatic veins or washout of contrast from the unopacified hepatic venous blood is looked for in order to identify the position of the hepatic veins.

**Figure 2:**
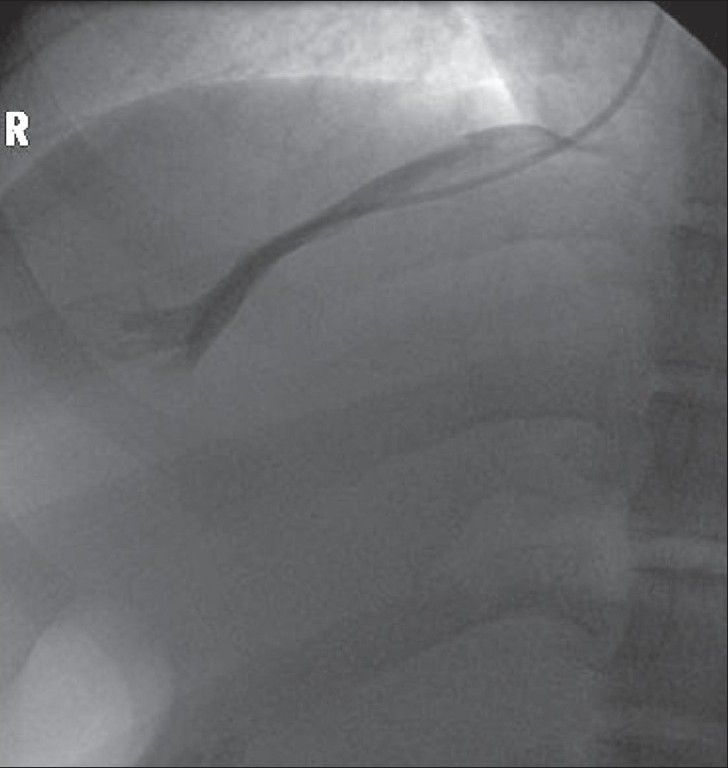
Right hepatic venogram

The catheter should be advanced along the main RHV, which will be oriented along the axis of the ribs. If the RHV cannot be cannulated, the middle or left hepatic veins can be tried. A hepatic venogram is performed by injecting 5 - 10 ml of contrast [Figure 2]. The venogram is essential to confirm that the catheter is in the hepatic vein. Rarely, the right renal vein can be mistaken for the RHV.

### Passing the stiffening cannula (14G; 50.5 cm) along with 7F sheath (49 cm)

The catheter is then exchanged for a TJLB stiffening cannula-7F sheath assembly. The stiffening cannula provides support and guidance for the Quick-Core biopsy needle. The directional arrow on the hub indicates the gentle curve towards the tip of the cannula.

The tip of the cannula is preferably placed in the middle third of the RHV. Any difficulty encountered is usually at the IVC-RHV junction. In such situations, one of the following, or a combination, would help:

- Changing the 0.035 inch guide wire to a stiff wire like a 0.035 inch Amplatz guide wire
- A coaxial 5F catheter (which comes in the LAPS set) leading the cannula and modifying the angle of the cannula.
- Attempting passage of the cannula in different phases of respiration (e.g., deep inspiration) may be of help.
- Gentle rotation of the cannula anteroposteriorly on the right side at the hepatic venous confluence may be helpful, since the RHV may not be in a true coronal plane.
- Changing the curve of the cannula manually outside the patient to match the curve on the wire at IVC-RHV junction.

### Biopsy

The Tru-Cut (Quick-Core) biopsy needle needs to be loaded/prepared outside by pulling back the plunger until a firm click is felt. The needle is passed through the stiffening cannula and the tip is projected just beyond the cannula in the middle third of the RHV. The peripheral one-third of the hepatic vein is better avoided so as to reduce the chances of a transcapsular puncture. The cannula has to be turned anteriorly if it is in the RHV and towards the right if it is in the middle hepatic vein (MHV); this helps in wedging the cannula against the liver parenchyma as well as in directing the needle towards the region where there is more liver tissue. Breath-holding is important at the time of biopsy to minimize any injury to the liver.

The needle has to be pulled into the stiffening cannula soon after the biopsy. Subsequently, the needle is pulled out of the cannula and the specimen is collected. The cannula has to be kept closed in between biopsies, either by the Check-Flo valve or by a syringe.

In general, three passes are sufficient for histopathology. Additional samples may be required for iron/copper estimation (dry weight) and culture and sensitivity. If the sample size is inadequate, one must make sure that the needle is wedged properly. Sometimes only fragmented pieces may be obtained due to the nodular nature of the diseased liver.

### Immediate post-biopsy care

A hepatic venogram is optional. If the procedure was eventful, a check venogram must be performed to exclude contrast extravasations. As soon as the cannula is pulled out, the patient can be made to sit up or may be kept in a semirecumbent position. Hemostasis at the puncture site can be obtained by manual compression.

### Post-biopsy (recovery room/ward)

The sitting/semirecumbent position may be ideal for patient nursing for the first 6 h following the procedure; the vital parameters - pulse and BP - are monitored two hourly for 6 h. Abdominal girth can also be monitored.

The average fluoroscopy time is 4 min. The mean duration of the procedure is 40 min and the radiation dose ranges from 0.5 - 1 mSv.[10]

### Special considerations

It may be relevant to obtain biopsy from the left lobe if there is gross volume redistribution in the liver. If none of the hepatic veins can be cannulated due to anatomical factors, a transcaval biopsy can be attempted. The intrahepatic location of the cannula must be confirmed prior to transcaval biopsy by transabdominal US. In case of situs inversus, the procedure can be performed in an anatomically reverse fashion.

Pediatric patients require general anesthesia. The adult TJLB cannula usually works for children also.

### Success rate

A technical success rate of 96.8% has been reported in a recent meta-analysis that included more than 7500 cases.[4] Inability to catheterize the RHV was the commonest reason (43.3%) for failure. Failure to access the IJV was more common when USG guidance was not used.

TJLB provides specimens which are qualitatively comparable to that obtained by PLB.[4]

### Complications

The complications are access site - related and cardiac or hepatic complications. The reported total complication rate is 7.1%. Mortality rates of 0.09% (adults) and 0.1% (children) have also been reported.[4] Mortality is due to hemorrhage from the liver or ventricular arrhythmia. Other complications included neck pain, hematoma in the neck, carotid artery puncture, pneumothorax, etc.

### Management of postprocedural bleeding

Significant hemorrhage requires immediate attention. Bleeding could either be into the peritoneal cavity or into the biliary tree. Intraperitoneal bleeding should be suspected if the patient experiences abdominal pain and distension; USG can help in the diagnosis. A venogram will be useful in such situations to identify the site of the bleeding, following which the bleeding site can be embolized.

If the bleeding is into the gastrointestinal tract, an endoscopy may be useful to differentiate hemobilia from variceal bleeding. A selective hepatic angiogram may reveal a hepatic artery pseudoaneurysm or biliary fistula, which would require embolization. Empirical embolisation of RHV branches may be considered if the blood loss is significant and the bleeding site cannot be located.

## Transjugular liver biopsy: Summary

**Table:** 

Indications	Ascites, deranged bleeding parameters in patients requiring liver biopsy
Principle	Transvenous biopsy, avoiding capsular puncture
Access	Right internal jugular
Needle	True-Cut 18G
Biopsy site	RHV
Success	97%
Complication	7.1%; may be at the access site or there may be cardiac or hepatic complications

**Table:** What to do

Proper selection of the cases	Make sure that liver biopsy is required and PLB is not possible
Adequate support from USG	To confirm the patency of hepatic veins whenever relevant
	For jugular access
	Transabdominal USG while doing biopsy of a small liver
Type of needle	Tru-Cut (Quick-Core)
Biopsy of a ‘stationary’ liver to minimize injury	Breath-holding
Wedge the cannula against liver parenchyma	To turn the cannula anterior in the RHV, turn towards the right from MHV
Back-up facilities to manage complications	Facilities for angiogram and embolization

**Table:** What not to do

Avoid carotid puncture	Patient can develop a neck hematoma, especially if the patient's bleeding parameters are deranged. A careful puncture under USG guidance can easily avoid this problem. Some people routinely use micropuncture to minimize the chances of hematoma by inadvertent carotid puncture.
Avoid arrhythmias	Minimize manipulation in the right atrium. It is mandatory to have facilities for treating arrhythmias and cardiac arrest. Usually the arrhythmia is transient.
Avoid air embolism	Air embolism can be fatal; do not leave any puncture needle/cannula open when its tip is inside a vein.
Avoid transcapsular puncture	It is possible that some of the punctures may be transcapsular in spite of all efforts to be strictly within the liver parenchyma. This may be due to less amount of liver tissue in front of the RHV, stretching of the hepatic vein, or entry of the cannula into the parenchyma, etc. A small liver poses the most problems. Gross ascites may be a compounding factor in the wrong estimation of the liver size on fluoroscopy. Liver size should be assessed based on the hepatic venography and not by a casual visual estimation of the distance from the midline to the lateral trunk wall!

## Conclusion

TJLB is becoming more relevant in the management of diffuse liver parenchymal diseases as it provides a safe technique for liver biopsy in patients in whom PLB is not feasible. TJLB with the Quick-Core biopsy needle is safe and effective in patients in whom the percutaneous route is contraindicated due to coagulopathy or ascites. Earlier, the quality of the specimen obtained was deemed to be suboptimal, but this was due to the use of the aspiration biopsy needle; adequate specimens can be obtained if 18G Quick-Core biopsy needles are used.

## References

[1] Colapinto RF (1985). Transjugular biopsy of the liver. Clin Gastroenterol.

[2] Weiner M, Hanafee WN (1970). A review of transjugular cholangiography. Radiol Clin North Am.

[3] Sada PN, Ramakrishna B, Thomas CP, Govil S, Koshi T, Chandy G (1997). Transjugular liver biopsy: A comparison of aspiration and trucut techniques. Liver.

[4] Kalambokis G, Manousou P, Vibhakorn S, Marelli L, Cholongitas E, Senzolo M (2007). Transjugular liver biopsy--indications, adequacy, quality of specimens, and complications: A systematic review. J Hepatol.

[5] Bruzzi JF, O'Connell MJ, Thakore H, O'Keane C, Crowe J, Murray JG (2002). Transjugular liver biopsy: Assessment of safety and efficacy of the Quick-Core biopsy needle. Abdom Imaging.

[6] Ishikawa T, Kamimura H, Tsuchiya A, Togashi T, Watanabe K, Seki K (2006). Comparison of a new aspiration needle device and the Quick-Core biopsy needle for transjugular liver biopsy. World J Gastroenterol.

[7] Banares R, Alonso S, Catalina MV, Casado M, Rincon D, Salcedo M (2001). Randomized controlled trial of aspiration needle versus automated biopsy device for transjugular liver biopsy. J Vasc Interv Radiol.

[8] Yavuz K, Geyik S, Barton RE, Petersen B, Lakin P, Keller FS (2007). Transjugular liver biopsy via the left internal jugular vein. J Vasc Interv Radiol.

[9] Khosa F, McNulty JG, Hickey N, O'Brien P, Tobin A, Noonan N (2003). Transvenous liver biopsy via the femoral vein. Clin Radiol.

[10] Mammen T, Keshava SN, Eapen CE, Raghuram L, Moses V, Gopi K (2008). Transjugular liver biopsy: A retrospective analysis of 601 cases. J Vasc Interv Radiol.

